# A prospective study to evaluate febrile neutropenia incidence in patients receiving pegfilgrastim on-body injector vs other choices

**DOI:** 10.1007/s00520-022-07226-9

**Published:** 2022-06-22

**Authors:** Robert M. Rifkin, Jeffrey Crawford, Reshma L. Mahtani, David C. Dale, Mohit Narang, William W. MacLaughlin, Chanh Huynh, Prasad L. Gawade, Sandra Lewis, Lucy DeCosta, Tatiana Lawrence, Rajesh Belani

**Affiliations:** 1 US Oncology Hematology Research, Rocky Mountain Cancer Centers – Midtown, 1800 Williams Street, Suite 200, Denver, CO 80218 USA; 2 Duke University School of Medicine, Durham, NC USA; 3 Division of Hematology/Oncology, Sylvester Comprehensive Cancer Center, University of Miami Health System, Deerfield Beach, FL USA; 4 Department of Medicine, University of Washington, Seattle, WA USA; 5 US Oncology Research, Maryland Oncology Hematology, P.A, Columbia, MD USA; 6 Riverside Health System, Chesapeake, VA USA; 7 Cancer Care Associates of York, York, PA USA; 8 Amgen Inc., Thousand Oaks, CA USA; 9 Global Biostatistical Science, Amgen Ltd, Cambridge, UK

**Keywords:** Febrile neutropenia, Pegfilgrastim, On-body injector (OBI)

## Abstract

**Purpose:**

We evaluated the incidence of febrile neutropenia (FN) and related clinical outcomes among patients treated with myelosuppressive chemotherapy for nonmyeloid malignancies who received pegfilgrastim on-body injector (OBI) or other options (Other) for FN prophylaxis.

**Methods:**

In this prospective observational study, adult patients with breast, prostate, or lung cancer, or non-Hodgkin lymphoma at risk for FN were stratified into subgroups based on FN prophylaxis used in the first chemotherapy cycle: pegfilgrastim OBI vs Other (pegfilgrastim or biosimilar pegfilgrastim prefilled syringe, daily filgrastim, or no granulocyte colony–stimulating factor [G-CSF]) for up to 4 planned chemotherapy cycles.

**Results:**

This US study enrolled 2575 eligible patients (OBI, 1624; Other, 951). FN incidence was lower in the OBI group (6.4% [95% CI, 5.2–7.6%]) than in the Other group (9.4% [7.5–11.2%]), with a relative risk (RR) of 0.66 (0.47–0.91; *p* = .006). A decreased risk of dose delays among patients receiving pegfilgrastim OBI vs Other was observed (RR for ≥ 5 days: 0.64 [0.42–0.96], *p* = .023; RR for ≥ 7 days: 0.62 [0.40–0.91], *p* = .016). Adherence, defined as G-CSF support for all chemotherapy cycles, was 94.0% (92.9–95.2%) in the OBI group compared with 58.4% (55.2–61.5%) in the Other group. Compliance with pegfilgrastim, defined as administration the day after chemotherapy, was 88.3% in the OBI group and 48.8% in the prefilled syringe group.

**Conclusion:**

Patients receiving pegfilgrastim OBI had a lower incidence of FN compared with those receiving alternatives. The OBI was associated with improved adherence to and compliance with clinically recommended G-CSF prophylaxis.

**Supplementary Information:**

The online version contains supplementary material available at 10.1007/s00520-022-07226-9.

## Introduction

Febrile neutropenia (FN), an oncologic emergency, can occur in adult patients with nonmyeloid malignancies receiving myelosuppressive chemotherapy regimens. FN is associated with increased hospitalization and healthcare costs and worse clinical outcomes, and can result in life-threatening complications [1–4]. Additionally, chemotherapy dose reductions and delays resulting from FN can reduce treatment effectiveness [5–7]. For over a decade, granulocyte colony–stimulating factor (G-CSF) agents have been indicated to prevent FN. These agents include short-acting G-CSFs and long-acting G-CSFs. Primary prophylaxis with G-CSF is more effective in preventing FN and FN-related complications than secondary prophylaxis or prophylaxis with antibiotics [3, 8–14]. Between filgrastim, pegfilgrastim, and lenograstim, a systematic review of the literature suggests that pegfilgrastim is most effective in reducing FN incidence [15].

Clinical guidelines and product prescribing information recommend the administration of a G-CSF 24 h after chemotherapy. Patients receiving pegfilgrastim the day after chemotherapy have less severe and shorter suppression of absolute neutrophil count (ANC) than those receiving pegfilgrastim the same day as chemotherapy [16, 17]. The Onpro® on-body injector (OBI) facilitates the time-released administration of pegfilgrastim the day after chemotherapy. The OBI is applied to patients on the last day of chemotherapy, thereby eliminating the need to return to the clinic the next day, which improves patient-centered care.

The NCCN guidelines recommend G-CSF support for patients receiving myelosuppressive chemotherapy regimens with a high risk (> 20%) for FN or an intermediate risk (10–20%) plus ≥ 1 patient risk factor for FN [18]. Despite guidelines on the use of G-CSF, FN continues to be a significant complication of cancer chemotherapy in the USA, with approximately 200,000 FN-related hospitalizations among adult cancer patients each year between 2016 and 2018 (data on file, Amgen; 2021). In a recent retrospective study of 22,868 patients with nonmyeloid malignancies, the proportions of patients receiving G-CSF in the first cycle were 76.1% and 26.4% among patients receiving regimens with a high risk for FN and an intermediate risk plus ≥ 1 risk factor for FN, respectively [19]. The reasons for the continued high incidence of FN are multifactorial, likely relating to inadequate G-CSF use, challenging healthcare economics, and the logistical burden associated with G-CSF therapy.

Real-world clinical data on whether the pegfilgrastim OBI reduces FN risk and improves patient adherence to G-CSF support and compliance with G-CSF prophylaxis are limited. The interpretation of retrospective claims-based data is challenging due to the lack of a designated International Classification of Diseases (ICD)-9/ICD-10 code for FN and the lack of a separate Healthcare Common Procedure Coding System (HCPCS) code that differentiate between pegfilgrastim OBI and prefilled syringe use. Therefore, to evaluate clinical outcomes and potential benefits of OBI use compared with other physician choice options for FN prophylaxis, a prospective, multicenter, observational study of adult patients with nonmyeloid malignancies receiving chemotherapy and at high risk for FN was conducted.

## Methods

### Study population

An internal review board approved the prespecified study protocol before initiating recruitment. The investigators obtained informed consent from each participant or each participant’s legally acceptable representative. The study prospectively recruited eligible adult patients diagnosed with non-Hodgkin lymphoma (NHL) or breast, lung, or prostate cancer with ≥ 4 anticipated chemotherapy cycles; with life expectancy > 6 months; and starting myelosuppressive chemotherapy regimens administered every 3 to 4 weeks with a high FN risk (> 20%) or an intermediate FN risk (10–20%) plus ≥ 1 patient risk factor per the NCCN guidelines version in use at the time of the protocol development.

Patients were classified into 2 groups, curative or palliative treatment intent, and then categorized by FN prophylaxis based on the first chemotherapy cycle: pegfilgrastim (Neulasta® Onpro®; Amgen Inc., Thousand Oaks, CA) OBI vs other options for FN prophylaxis (treating physicians had the discretion to select pegfilgrastim or biosimilar pegfilgrastim prefilled syringe, daily filgrastim, or no G-CSF) for up to 4 planned chemotherapy cycles. The study schema is depicted in Fig. S1. The group assignment would remain the same from the first cycle, regardless of changes to prophylaxis in subsequent cycles. Each patient was followed up from enrollment until the earliest occurrence of death, discontinuation of chemotherapy regimen before 4 cycles, withdrawal of consent, loss to follow-up, or end of the study. Patients were enrolled between November 7, 2018, and April 9, 2020.

### Endpoints

The primary endpoint was the incidence of FN. FN was defined as ANC < 1000 × 10^6^/L and one of the following occurring within 24 h of decreased ANC: temperature > 38 °C, use of specific oral antibiotics, or use of any intravenous antibiotics. Secondary endpoints included chemotherapy delivery, adherence to G-CSF therapy, and compliance with pegfilgrastim therapy. Chemotherapy delivery was defined as dose delays of ≥ 5 and ≥ 7 days (extension of the time between planned chemotherapy cycles on or before cycle 4) and/or dose reductions of ≥ 15% (decrease in the dose of planned chemotherapy on or before cycle 4). Adherence was defined as G-CSF support for all chemotherapy cycles regardless of the timing of G-CSF administration. G-CSF dosing per cycle included 1 pegfilgrastim OBI, 1 pegfilgrastim prefilled syringe, 1 biosimilar pegfilgrastim prefilled syringe, or 10 short-acting G-CSF injections. Compliance with pegfilgrastim therapy was defined as receiving pegfilgrastim on the day after the last day of chemotherapy administration at every cycle in which pegfilgrastim was administered. Physician-reported failure of the OBI device was defined as a citation on the G-CSF administration form as the reason for a zero or partial dose, dose delay, or dose interruption.

### Sample size estimation

The study was designed as an estimation study; however, a sample size of 2220 patients was targeted in each of the OBI and other physician choice options (Other) group for the curative intent cohort to allow nonoverlapping 95% confidence intervals (CIs) for the estimation of FN incidence. The assumptions for 95% CIs were based on observed FN incidences of 7.5% and 10% in patients receiving pegfilgrastim OBI and Other, respectively. A maximum of 500 patients were planned in each of the 2 groups of the palliative intent cohort. Due to the COVID-19 pandemic, enrollment was halted in April 2020. The COVID-19 pandemic and the interim NCCN COVID-19–related recommendations [20] likely would impact the cancer treatment landscape and potentially increase G-CSF use for intermediate-risk chemotherapies, confounding the outcomes. In April 2020 [20], the NCCN Hematopoietic Growth Factors Panel issued COVID-19–related interim recommendations suggesting G-CSF support be given to patients treated with regimens that have a high to intermediate FN risk without consideration of risk factors. These changes would have introduced bias into the study if it had continued, likely rendering the assumptions used in the study design and the projected recruitment timelines at risk to commit as planned. After careful evaluation of the results of the prespecified second interim analysis of 2000 enrolled patients, the impact of the COVID-19 pandemic on the study, and the extensive time investment required to complete study enrollment, the sponsor and the steering committee decided to close patient enrollment before reaching the target numbers.

### Statistical analysis

To determine the relative risks (RRs) of FN in patients receiving pegfilgrastim OBI vs Other, FN incidence was first adjusted by a standardized log-binomial model to control for confounding factors [21]. The covariates included tumor type, age, sex, health plan/insurance, tobacco use, Eastern Cooperative Oncology Group (ECOG) performance status, history of any other malignancy except nonmelanoma skin cancer, history of surgery, chemotherapy, or radiation therapy within 6 months before enrollment, number of comorbidities, bone marrow involvement, baseline laboratory measurements (hemoglobin, ANC, white blood cell, platelets, lactate dehydrogenase, and alkaline phosphatase), antibiotic use prior to initiation of chemotherapy in cycle 1, and FN risk of chemotherapy regimen. *P*-values were calculated post hoc. Associated 95% CIs for RRs were calculated using a bootstrap method. Subgroup analyses for the incidence of FN among patients with curative or palliative intent, and for each specific tumor type were also performed. The Medical Dictionary for Regulatory Activities (MedDRA) version 22.1 was used to code all reported adverse events. Comorbidities were recorded at baseline (Table S1).

## Results

### Patient disposition and baseline characteristics

A total of 2715 patients from 144 sites in the USA were enrolled between November 7, 2018, and April 9, 2020. Of the 2575 eligible patients (Table S2), 2149 were treated with curative intent and 426 patients had a goal of palliative care. Demographics and baseline characteristics of the curative and palliative intent groups were described (Table S3). Because the number of patients with palliative intent enrolled was small and was not expected to impact the overall results, the analysis primarily focused on patients receiving pegfilgrastim OBI or other physician choice options (Other) without the classification of treatment intent as defined per the primary endpoint.

Patients enrolled faster in the OBI group than in the Other group (OBI, 1624; Other, 951). The two groups were generally comparable (Table 1). The patient population in this study was predominantly female (79.7%), which was consistent with breast cancer being the most common tumor type, although more patients with breast cancer received pegfilgrastim OBI (OBI, 73.6%; Other, 61.0%).

**Table 1:** Patient demographics and baseline characteristics

	On-body injector	Other physician choice	All patients
	(*n* = 1624)	(*n* = 951)	(*N* = 2575)
Sex—*n* (%)			
Male	271 (16.7)	251 (26.4)	522 (20.3)
Female	1353 (83.3)	700 (73.6)	2053 (79.7)
Age—years			
Median (IQR)	62 (52–70)	63 (54–70)	62 (53–70)
Tumor type—*n* (%)			
Breast	1196 (73.6)	580 (61.0)	1776 (69.0)
Non-Hodgkin lymphoma	236 (14.5)	177 (18.6)	413 (16.0)
Lung	132 (8.1)	132 (13.9)	264 (10.3)
Prostate	60 (3.7)	62 (6.5)	122 (4.7)
ECOG performance status—*n* (%)			
0–1	1561 (96.1)	905 (95.2)	2466 (95.8)
≥ 2	50 (3.1)	44 (4.6)	94 (3.7)
Missing	13 (0.8)	2 (0.2)	15 (0.6)
Number of comorbidities—*n* (%)			
> 2	325 (20.0)	195 (20.5)	520 (20.2)
≤ 2	1299 (80.0)	756 (79.5)	2055 (79.8)
History of any other malignancy^a^—*n* (%)			
Yes	97 (6.0)	95 (10.0)	192 (7.5)
No	1527 (94.0)	856 (90.0)	2383 (92.5)
Antibiotic use prior to initiation of chemotherapy—*n* (%)			
Yes	131 (8.1)	110 (11.6)	241 (9.4)
No	1493 (91.9)	841 (88.4)	2334 (90.6)
Prior surgery^b^—*n* (%)			
Yes	1250 (77.0)	601 (63.2)	1851 (71.9)
No	374 (23.0)	350 (36.8)	724 (28.1)
Prior chemotherapy^b^—*n* (%)			
Yes	7 (0.4)	8 (0.8)	15 (0.6)
No	1617 (99.6)	943 (99.2)	2560 (99.4)
Prior radiotherapy^b^—*n* (%)			
Yes	35 (2.2)	24 (2.5)	59 (2.3)
No	1589 (97.8)	927 (97.5)	2516 (97.7)

*ECOG*, Eastern Cooperative Oncology Group; *IQR*, interquartile range

^a^Excluding nonmelanoma skin cancer

^b^Within 6 months prior to study enrollment

Sixty-six percent of patients in the OBI group received chemotherapy regimens associated with a high FN risk compared with 52% in the Other group (Table 2). The top chemotherapy regimen with high risk for FN was the combination of docetaxel and cyclophosphamide, followed by the combination of docetaxel, carboplatin, trastuzumab, and pertuzumab. Among patients receiving chemotherapy regimens with intermediate risk, the most common regimen was the combination of rituximab, cyclophosphamide, doxorubicin, vincristine, and prednisone. The FN risk and chemotherapy regimens for patients with curative or palliative intent were shown (Table S4). All included regimens had at least a 3-week interval of dosing.

**Table 2:** Febrile neutropenia risk of chemotherapy regimens administered to patients

	On-body injector	Other physician choice	All patients
	(*n* = 1624)	(*n* = 951)	(*N* = 2575)
FN risk of chemotherapy regimen—*n* (%)			
High	1079 (66.4)	493 (51.8)	1572 (61.0)
Intermediate	545 (33.6)	458 (48.2)	1003 (39.0)
Chemotherapy regimen—*n* (%)			
High risk for FN (> 20%)			
TC	573 (35.3)	254 (26.7)	827 (32.1)
TCHP	392 (24.1)	182 (19.1)	574 (22.3)
TCH	82 (5.0)	32 (3.4)	114 (4.4)
TAC	23 (1.4)	9 (0.9)	32 (1.2)
R-da EPOCH	8 (0.5)	15 (1.6)	23 (0.9)
Dose-adjusted EPOCH	1 (< 0.1)	0 (0.0)	1 (< 0.1)
R-ICE	0 (0.0)	1 (0.1)	1 (< 0.1)
Intermediate risk for FN (10–20%)			
R-CHOP	163 (10.0)	89 (9.4)	252 (9.8)
Etoposide and carboplatin	70 (4.3)	70 (7.4)	140 (5.4)
AC	63 (3.9)	47 (4.9)	110 (4.3)
Docetaxel	59 (3.6)	71 (7.5)	130 (5.0)
Carboplatin and paclitaxel	59 (3.6)	55 (5.8)	114 (4.4)
Bendamustine and rituximab	53 (3.3)	71 (7.5)	124 (4.8)
AC → T	47 (2.9)	21 (2.2)	68 (2.6)
CHOP	10 (0.6)	1 (0.1)	11 (0.4)
TH	5 (0.3)	1 (0.1)	6 (0.2)
Cisplatin and docetaxel	4 (0.2)	4 (0.4)	8 (0.3)
Cabazitaxel	4 (0.2)	1 (0.1)	5 (0.2)
Paclitaxel	3 (0.2)	2 (0.2)	5 (0.2)
CMF classic	2 (0.1)	22 (2.3)	24 (0.9)
Cisplatin and etoposide	2 (0.1)	3 (0.3)	5 (0.2)
Carboplatin nab-paclitaxel	1 (< 0.1)	0 (0.0)	1 (< 0.1)

*AC*, doxorubicin, cyclophosphamide; *AC → T*, doxorubicin, cyclophosphamide → docetaxel; *CHOP*, cyclophosphamide, doxorubicin, vincristine, prednisone; *CMF*, cyclophosphamide, methotrexate, fluorouracil; *EPOCH*, etoposide, prednisone, vincristine, cyclophosphamide, doxorubicin; *FN*, febrile neutropenia; *ICE*, ifosfamide, carboplatin, etoposide; *R*, rituximab; *R-da EPOCH*, rituximab, dose-adjusted etoposide, prednisone, vincristine, cyclophosphamide, doxorubicin; *TAC*, docetaxel, doxorubicin, cyclophosphamide; *TC*, docetaxel, cyclophosphamide; *TCH*, docetaxel, carboplatin, trastuzumab; *TCHP*, docetaxel, carboplatin, trastuzumab, pertuzumab; *TH*, docetaxel, trastuzumab

### Incidence of febrile neutropenia

**Fig. 1:**
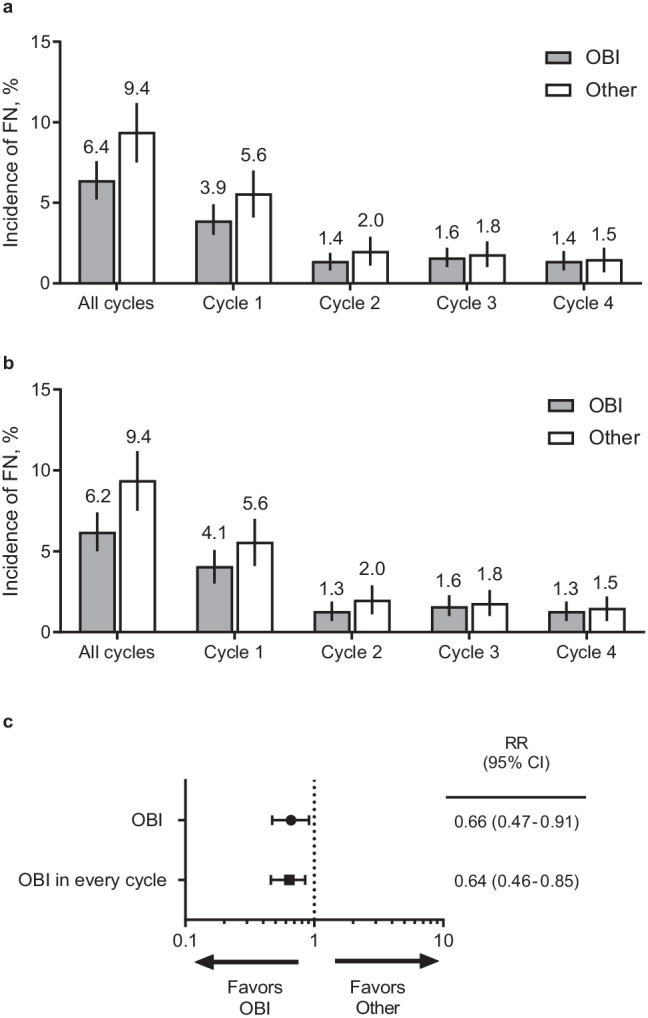
Incidence of febrile neutropenia. (**a**) FN incidence in patients receiving pegfilgrastim OBI vs other physician choice options. (**b**) FN incidence in patients receiving pegfilgrastim OBI in every cycle vs other physician choice options. (**c**) Relative risk of FN. Error bars denote 95% CIs. *CI*, confidence interval; *FN*, febrile neutropenia; *OBI*, on-body injector; *Other*, other physician choice options; *RR*, relative risk

The incidence of FN was lower among patients who received pegfilgrastim OBI compared with patients who received other options (6.4% [95% CI, 5.2–7.6%] for OBI [FN, *n* = 104; OBI, *n* = 1624] vs 9.4% [95% CI, 7.5–11.2%] for Other [FN, *n* = 89; Other, *n* = 951]) (Fig. 1a). The incidence of FN was lower in the OBI group than in the Other group across all cycles (3.9% vs 5.6%, 1.4% vs 2.0%, 1.6% vs 1.8%, and 1.4% vs 1.5% for cycles 1, 2, 3, and 4, respectively). In a subgroup analysis among patients who received pegfilgrastim OBI in every cycle, the FN incidence was 6.2% (FN, *n* = 90; OBI in every cycle, *n* = 1455; 95% CI, 5.0–7.4%), similar to the incidence in the overall OBI group (Fig. 1b). An FN risk reduction of 34% was observed in patients who received pegfilgrastim OBI (RR: 0.66 [95% CI, 0.47–0.91; *p* = 0.006]; Fig. 1c). The FN incidence in patients receiving G-CSF in at least 1 cycle (≥ 1 G-CSF) in the Other group was 10.0% (FN, *n* = 72; Other who received ≥ 1 G-CSF, *n* = 723; 95% CI, 7.8–12.1%) (Fig. S2a). The FN risk reduction among patients who received pegfilgrastim OBI in every cycle (RR: 0.64 [95% CI, 0.46–0.85]; *p* = 0.004) was similar to those receiving pegfilgrastim OBI (Fig. 1c). Regardless of the intent of treatment (curative or palliative), patients receiving pegfilgrastim OBI had a lower FN incidence than those receiving the alternatives (Fig. S2b). The FN incidence by tumor type (breast, NHL, lung, or prostate) ranged from 1.7 to 18.6% in the OBI groups and 3.2 to 16.4% in the Other groups (Fig. S2c).

### Chemotherapy delivery

**Fig. 2:**
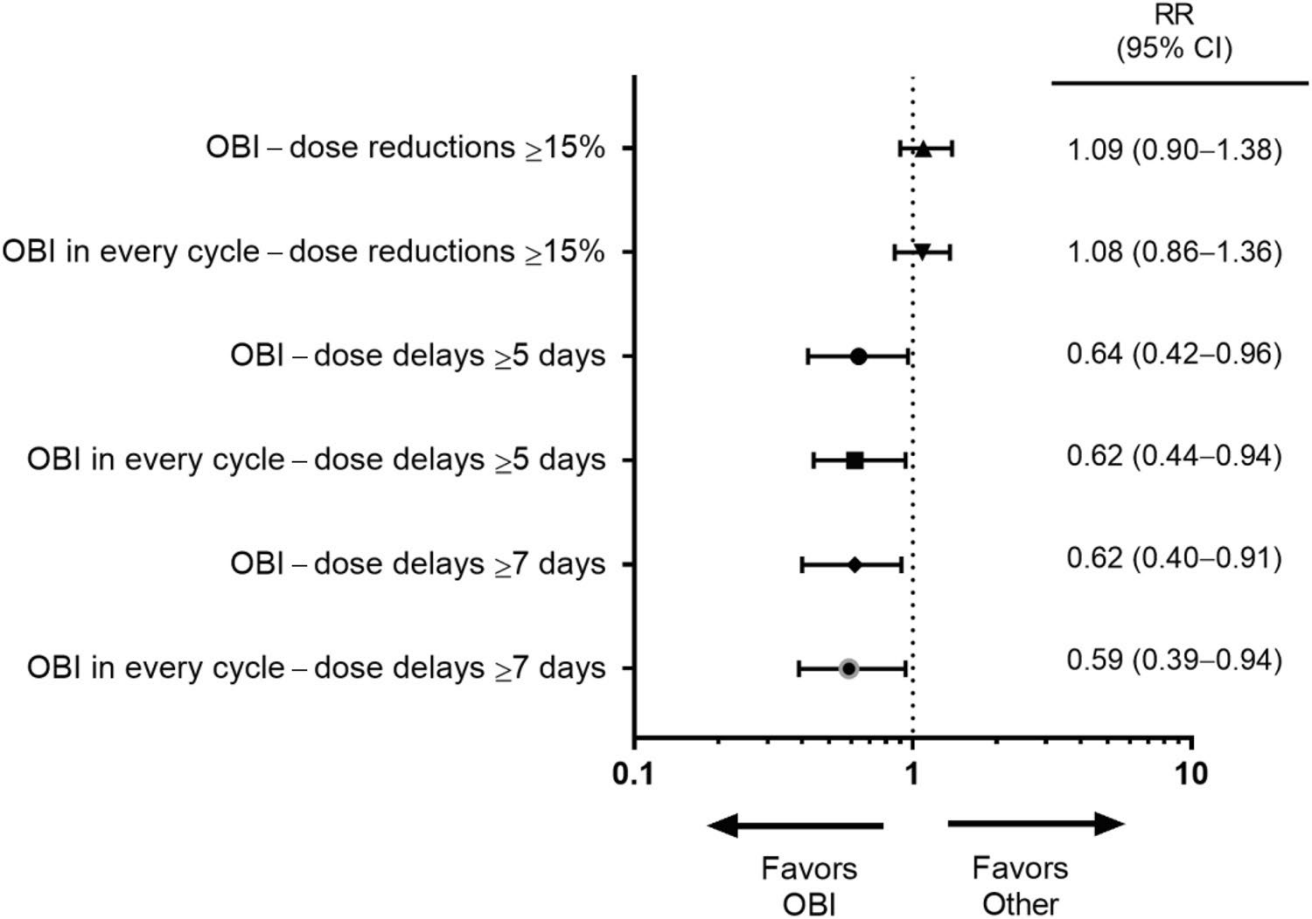
Relative risks of chemotherapy dose delays (≥ 5 and ≥ 7 days)/reductions (≥ 15%) in patients receiving pegfilgrastim OBI, and those receiving pegfilgrastim OBI in every cycle vs other physician choice options. Error bars denote 95% CIs. *CI*, confidence interval; *OBI*, on-body injector; *RR*, relative risk

The risks of chemotherapy dose delays of ≥ 5 and ≥ 7 days were lower in the OBI group (RR for ≥ 5 days: 0.64 [95% CI, 0.42–0.96; *p* = 0.023]; RR for ≥ 7 days: 0.62 [95% CI, 0.40–0.91]; *p* = 0.016) than in the Other group, which were comparable with patients who received pegfilgrastim OBI in every cycle (RR for ≥ 5 days: 0.62 [95% CI, 0.44–0.94; *p* = 0.017]; RR for ≥ 7 days: 0.59 [95% CI, 0.39–0.94]; *p* = 0.011; Fig. 2). There was no difference in chemotherapy dose reductions of ≥ 15% between the 2 groups (OBI vs Other, RR: 1.09; or OBI in every cycle vs Other, RR: 1.08; Fig. 2).

### Adherence and compliance

**Fig. 3:**
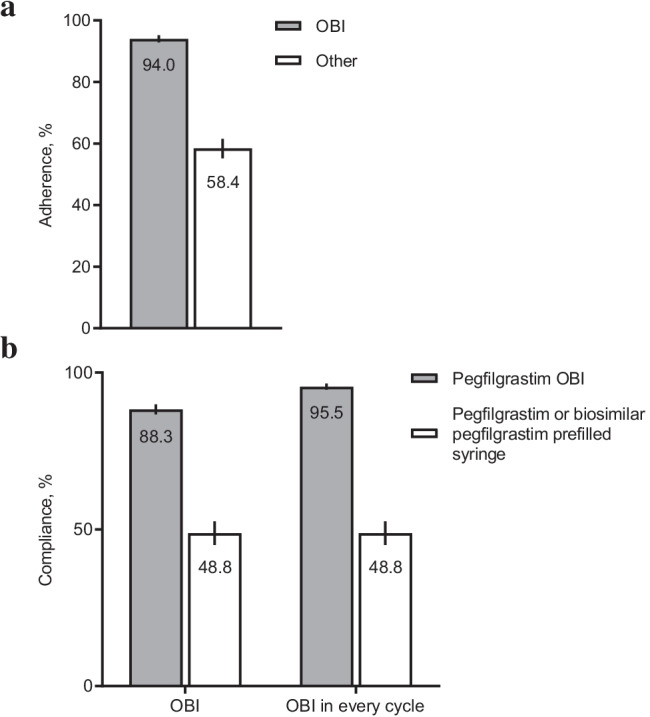
Adherence to G-CSF and compliance with pegfilgrastim. (**a**) Adherence and (**b**) Compliance in patients receiving pegfilgrastim OBI and in patients receiving pegfilgrastim OBI in every cycle. Error bars denote 95% CIs. *CI*, confidence interval; *G-CSF*, granulocyte colony–stimulating factor; *OBI*, on-body injector; *Other*, other physician choice options

Adherence, defined as G-CSF support for all chemotherapy cycles, was 94.0% (95% CI, 92.9–95.2%) in patients who received pegfilgrastim OBI compared with 58.4% (95% CI, 55.2–61.5%) in the Other group (Fig. 3a). Compliance (next-day administration) with pegfilgrastim was 88.3% (95% CI, 86.7–89.9%) in patients receiving pegfilgrastim OBI and 48.8% (95% CI, 45.0–52.6%) in patients receiving pegfilgrastim or biosimilar pegfilgrastim via a prefilled syringe (Fig. 3b). The compliance for patients who received pegfilgrastim OBI in every cycle was 95.5% (95% CI, 94.5–96.6%). Among patients who received pegfilgrastim OBI, only 6 of 6087 OBI administrations were reported as device failure (0.1%). Other common reasons for the noncompliance included not applying the injector on the last day of chemotherapy or modifying chemotherapy regimens, which constituted 4.5% of cycles.

### G-CSF utilization

The baseline characteristics for patients using OBI and those not receiving G-CSF prophylaxis are described (Table S5). Patients in the OBI group were more likely to be female and receive treatment with curative intent and a chemotherapy regimen with a high risk for FN. A small fraction of patients in the OBI group switched to other options (prefilled syringe, *n* = 80 [4.9%]; short-acting G-CSF, *n* = 20 [1.2%]; no G-CSF, *n* = 80 [4.9%]; Table 3). In the Other group, about two-thirds (64%) of patients received pegfilgrastim via a prefilled syringe and one-third (33%) of patients did not receive any G-CSF in at least 1 cycle and 24% in any cycle even though they were clinically indicated to receive G-CSF prophylaxis. Interestingly, the proportion of patients in the Other group not receiving any G-CSF support declined over the course of the study (from 30.9% in cycle 1 to 24.4% in cycle 4).

**Table 3:** G-CSF type in first cycle or across all cycles in patients receiving other physician choice options

	On-body injector	Other physician choice
	(*n* = 1624)	(*n* = 951)
G-CSF utilization in cycle 1—*n* (%)		
OBI	1624 (100.0)	0 (0.0)
Pegfilgrastim or biosimilar pegfilgrastim prefilled syringe	0 (0.0)	587 (61.7)
Short-acting G-CSF	0 (0.0)	70 (7.4)
No G-CSF	0 (0.0)	294 (30.9)
G-CSF utilization in all cycles^a^—*n* (%)		
OBI	1624 (100.0)	103 (10.8)
Pegfilgrastim or biosimilar pegfilgrastim prefilled syringe	80 (4.9)	610 (64.1)
Short-acting G-CSF^b^	20 (1.2)	98 (10.3)
No G-CSF	80 (4.9)	314 (33.0)

*G-CSF*, granulocyte colony–stimulating factor; *OBI*, on-body injector

^a^Categories are not mutually exclusive

^b^121 patients were administered short-acting G-CSFs via a prefilled syringe, and 2 patients received short-acting G-CSFs from a vial, accounting for less than 0.1% of the study population (*n* = 2575)

### Safety

A total of 9665 chemotherapy cycles were administered across both groups. The most common adverse events reported in the OBI and Other groups were anemia and bone pain (Table S6). The adverse events in the safety analysis set (OBI, *n* = 1624; Other, *n* = 935) were comparable between the 2 groups and to those that were reported previously in other G-CSF clinical studies [13, 22].

## Discussion

The clinical benefits of filgrastim and pegfilgrastim to prevent FN among cancer patients receiving myelosuppressive cytotoxic chemotherapy have been well established and supported by clinical guidelines [18]. This study is the largest prospective observational study to evaluate clinical outcomes in patients receiving pegfilgrastim through an OBI compared with other physician choice options for FN prophylaxis. Physicians have a variety of treatment options available to them for FN prophylaxis. The OBI group, in which patients received pegfilgrastim 27 h after chemotherapy, represented the homogenous comparator. The observed FN incidence in our study is consistent with that reported in prior studies. In 2 pivotal pegfilgrastim studies, the FN incidence in patients receiving filgrastim was 15–18% compared with 9–10% in those receiving pegfilgrastim across a variety of tumor types [22, 23].

OBI use was associated with a lower incidence of FN in patients receiving chemotherapy vs those receiving other FN prophylaxis options. The lower incidence with pegfilgrastim OBI is especially notable given that a higher proportion of patients in the OBI group were on high-FN-risk regimens, and consistent regardless of treatment intent (curative or palliative) and tumor type apart from NHL. However, the results from NHL, prostate cancer, and lung cancer subgroup analyses should be interpreted with caution given the small number of patients enrolled in each group.

To further examine OBI use, we evaluated patients within the OBI group who received OBI in every cycle. The FN incidence in these patients was 6.2%, with a 36% FN risk reduction in comparison to patients in the Other group (9.4%). When patients in the OBI group were compared with those who received ≥ 1 G-CSF in the Other group, the absolute difference in FN incidence was 3.6%, which was comparable to that observed between the OBI and Other groups. The exclusion of patients who did not receive G-CSF prophylaxis did not influence the FN incidence in the Other group.

Pegfilgrastim OBI also supports on-time chemotherapy delivery. Chemotherapy dose delays, reductions, or both may affect dose intensity, reducing the chance of patients achieving an optimal treatment response. Full-dose chemotherapy on schedule is associated with improved outcomes, especially for patients with early-stage breast cancer and NHL [5, 24–26]. Patients receiving pegfilgrastim OBI had a 41% lower risk of chemotherapy dose delays than those receiving other options for FN prophylaxis. However, there was no difference in dose reductions between both groups. The higher risk of dose delays observed in the Other group may have resulted from a higher proportion of patients experiencing FN.

All patients in this study met the criteria to receive G-CSF agents based on NCCN guideline recommendations. However, 33% of patients in the Other group did not receive G-CSF support in at least 1 cycle and 24% of patients in the Other group did not receive G-CSF support in any cycle. Two noteworthy differences in baseline characteristics were observed between patients using OBI and those not receiving G-CSF prophylaxis; most of the patients without G-CSF prophylaxis were treated with chemotherapy regimens with intermediate risk for FN albeit having at least 1 risk factor, and unlike patients in the OBI group, breast cancer was not the primary tumor type. The underuse of G-CSF is consistent with that observed in prior studies reporting suboptimal use of G-CSF [19, 27, 28]. In a retrospective observational study that evaluated patients from a commercial administrative database, 76% of breast cancer patients treated with high-FN-risk chemotherapy received G-CSF in the first cycle [19]. In another study that evaluated Medicare patients, 74% of breast cancer patients on high-FN-risk chemotherapy received G-CSF in the first cycle [28].

Although pegfilgrastim should not be administered until the day after chemotherapy according to the FDA-approved regimen, there has been an ongoing interest in evaluating administration on the same day as chemotherapy [29, 30]. Logistic concerns (e.g., scheduling and travel) and convenience continue to evoke interest in same-day administration. However, same-day administration is not as effective as next-day administration. In a recent and large (65,000 patients, 261,000 cycles) retrospective cohort study, same-day administration in patients with breast cancer and NHL aged ≥ 65 years on high- or intermediate-FN-risk regimens was associated with significantly higher rates of FN vs dosing on days 1–3 after chemotherapy (11.4% vs 8.4%, *p* < 0.001) [31]. Similarly, another recent systematic review with meta-analysis showed a significantly higher FN rate in patients administered same-day prophylaxis compared with those receiving next-day prophylaxis both in the first cycle (odds ratio [OR] = 2.56, 95% CI, 1.19–5.48, *p* = 0.02) and across all cycles (OR = 1.54, 95% CI, 1.29–1.84, *p* < 0.00001) [32]. At the 2021 American Society of Clinical Oncology (ASCO) Annual Meeting, 3 small (< 120 patients) retrospective analyses evaluated same-day administration of prefilled syringe all studies showed increased rates of FN with same-day administration [33–35]. Pegfilgrastim, when used as indicated, decreases chemotherapy-induced FN. Based on the results of this study, pegfilgrastim OBI could facilitate adherence to the recommended guidelines, as demonstrated by the greater compliance rate in patients using an OBI (95.5% for OBI in every cycle vs 48.8% for prefilled syringe). In a recent retrospective study, better adherence was also observed in patients with breast cancer receiving high-FN-risk regimens and starting primary prophylactic pegfilgrastim via an OBI; 60.4% (95% CI, 57.2–63.6%) of patients initiating via an OBI and 51.9% (95% CI, 48.0–55.8%) initiating via a prefilled syringe completed all their cycles [19]. The noncompliance rate of 4.5% in the OBI group was primarily related to changes in chemotherapy regimens and not to OBI performance as there was a device failure rate of only 0.1% in the study. Additionally, a 3-year postmarketing commitment in the EU reported that the OBI device reliability (*N* = 27,666 distributed devices) was greater than 99% [36].

### Limitations

Several limitations should be considered for the study. The unforeseen COVID-19 pandemic resulted in the early cessation of the study before reaching the target sample size. Selection bias may have been introduced because of the inability to evaluate the FN risk among patients lost to follow-up after study enrollment. Confounding by indication of deliberate effect may have lowered the FN incidence for the Other group due to physicians’ intention to not provide G-CSF prophylaxis to relatively healthier patients or to those at lower risk of FN per their individual assessment of the patient. Standardized log-binomial regression was applied in the analysis to control confounding, but the model did not account for censoring events that occurred during the follow-up period. It is conceivable that patients in one group may have a shorter overall at-risk period for developing FN than those in the other group because of censoring events.

## Conclusions

This is the largest prospective observational study yet conducted examining the impact of physician’s choice of G-CSF support in the real world. Cancer patients treated with myelosuppressive chemotherapy receiving pegfilgrastim OBI had a lower rate of FN than those receiving other prophylactic strategies. The lower incidence of FN associated with the OBI may be related to the greater adherence and compliance with G-CSF support seen in this study.

## Supplementary Information

Below is the link to the electronic supplementary material.

Supplementary file1 (DOCX 114 KB)

## Data Availability

Qualified researchers may request data from Amgen clinical studies. Complete details are available at http://www.amgen.com/datasharing.

## References

[1] Caggiano V, Weiss RV, Rickert TS, Linde-Zwirble WT (2005). Incidence, cost, and mortality of neutropenia hospitalization associated with chemotherapy. Cancer.

[2] Kuderer NM, Dale DC, Crawford J, Cosler LE, Lyman GH (2006). Mortality, morbidity, and cost associated with febrile neutropenia in adult cancer patients. Cancer.

[3] Kuderer NM, Dale DC, Crawford J, Lyman GH (2007). Impact of primary prophylaxis with granulocyte colony-stimulating factor on febrile neutropenia and mortality in adult cancer patients receiving chemotherapy: a systematic review. J Clin Oncol.

[4] Li S, Liu J, Bowers C, Garawin T, Kim C, Bensink ME, Chandler DB (2020). Febrile neutropenia-related care and associated costs in elderly patients with breast cancer, lung cancer, or non-Hodgkin lymphoma. Support Care Cancer.

[5] Lyman GH, Dale DC, Crawford J (2003). Incidence and predictors of low dose-intensity in adjuvant breast cancer chemotherapy: a nationwide study of community practices. J Clin Oncol.

[6] Lyman GH, Dale DC, Culakova E, Poniewierski MS, Wolff DA, Kuderer NM, Huang M, Crawford J (2013). The impact of the granulocyte colony-stimulating factor on chemotherapy dose intensity and cancer survival: a systematic review and meta-analysis of randomized controlled trials. Ann Oncol.

[7] Lyman GH, Dale DC, Tomita D, Whittaker S, Crawford J (2013). A retrospective evaluation of chemotherapy dose intensity and supportive care for early-stage breast cancer in a curative setting. Breast Cancer Res Treat.

[8] Biron P, Ray-Coquard I, Cesne AL, Dussart S, Goilliot C, Bachelot T, Thyss A, Gilles E, Chabaud S, Blay J (2006). ELYPSE 2: a prospective randomized trial comparing filgrastim (G-CSF) in primary and secondary prophylaxis in patients (pts) at high risk for febrile neutropenia (FN). J Clin Oncol.

[9] Gerlier L, Lamotte M, Awada A, Bosly A, Bries G, Cocquyt V, Focan C, Henry S, Lalami Y, Machiels JP, Mebis J, Straetmans N, Verhoeven D, Somers L (2010). The use of chemotherapy regimens carrying a moderate or high risk of febrile neutropenia and the corresponding management of febrile neutropenia: an expert survey in breast cancer and non-Hodgkin’s lymphoma. BMC Cancer.

[10] Kuderer NM (2011). Meta-analysis of randomized controlled trials of granulocyte colony-stimulating factor prophylaxis in adult cancer patients receiving chemotherapy. Cancer Treat Res.

[11] Madan J, Stevenson MD, Cooper KL, Ades AE, Whyte S, Akehurst R (2011). Consistency between direct and indirect trial evidence: is direct evidence always more reliable?. Value Health.

[12] Morrison VA, Wong M, Hershman D, Campos LT, Ding B, Malin J (2007). Observational study of the prevalence of febrile neutropenia in patients who received filgrastim or pegfilgrastim associated with 3–4 week chemotherapy regimens in community oncology practices. J Manag Care Pharm.

[13] Vogel CL, Wojtukiewicz MZ, Carroll RR, Tjulandin SA, Barajas-Figueroa LJ, Wiens BL, Neumann TA, Schwartzberg LS (2005). First and subsequent cycle use of pegfilgrastim prevents febrile neutropenia in patients with breast cancer: a multicenter, double-blind, placebo-controlled phase III study. J Clin Oncol.

[14] Weycker D, Malin J, Kim J, Barron R, Edelsberg J, Kartashov A, Oster G (2009). Risk of hospitalization for neutropenic complications of chemotherapy in patients with primary solid tumors receiving pegfilgrastim or filgrastim prophylaxis: a retrospective cohort study. Clin Ther.

[15] Cooper KL, Madan J, Whyte S, Stevenson MD, Akehurst RL (2011). Granulocyte colony-stimulating factors for febrile neutropenia prophylaxis following chemotherapy: systematic review and meta-analysis. BMC Cancer.

[16] Amgen Inc. (2020) Neulasta Prescribing Information. https://www.pi.amgen.com/-/media/Project/Amgen/Repository/pi-amgen-com/Neulasta/neulasta_pi_hcp_english.pdf

[17] Li Y, Klippel Z, Shih X, Wang H, Reiner M, Page JH (2016). Trajectory of absolute neutrophil counts in patients treated with pegfilgrastim on the day of chemotherapy versus the day after chemotherapy. Cancer Chemother Pharmacol.

[18] NCCN clinical practice guidelines in oncology hematopoietic growth factors version 2.2020. Available at: NCCN.org. Accessed January 7, 2021

[19] Gawade PL, Li S, Henry D, Smith N, Belani R, Kelsh MA, Bradbury BD (2020). Patterns of granulocyte colony-stimulating factor prophylaxis in patients with cancer receiving myelosuppressive chemotherapy. Support Care Cancer.

[20] NCCN Short-term recommendations specific to issues with COVID-19. Available at: NCCN.org. Accessed January 7, 2021

[21] Richardson DB, Kinlaw AC, MacLehose RF, Cole SR (2015). Standardized binomial models for risk or prevalence ratios and differences. Int J Epidemiol.

[22] Green MD, Koelbl H, Baselga J, Galid A, Guillem V, Gascon P, Siena S, Lalisang RI, Samonigg H, Clemens MR, Zani V, Liang BC, Renwick J, Piccart MJ (2003). A randomized double-blind multicenter phase III study of fixed-dose single-administration pegfilgrastim versus daily filgrastim in patients receiving myelosuppressive chemotherapy. Ann Oncol.

[23] Holmes FA, O’Shaughnessy JA, Vukelja S, Jones SE, Shogan J, Savin M, Glaspy J, Moore M, Meza L, Wiznitzer I, Neumann TA, Hill LR, Liang BC (2002). Blinded, randomized, multicenter study to evaluate single administration pegfilgrastim once per cycle versus daily filgrastim as an adjunct to chemotherapy in patients with high-risk stage II or stage III/IV breast cancer. J Clin Oncol.

[24] Chirivella I, Bermejo B, Insa A, Pérez-Fidalgo A, Magro A, Rosello S, García-Garre E, Martín P, Bosch A, Lluch A (2009). Optimal delivery of anthracycline-based chemotherapy in the adjuvant setting improves outcome of breast cancer patients. Breast Cancer Res Treat.

[25] Denduluri N, Patt DA, Wang Y, Bhor M, Li X, Favret AM, Morrow PK, Barron RL, Asmar L, Saravanan S, Li Y, Garcia J, Lyman GH (2015). Dose delays, dose reductions, and relative dose intensity in patients with cancer who received adjuvant or neoadjuvant chemotherapy in community oncology practices. J Natl Compr Canc Netw.

[26] Lyman GH (2009). Impact of chemotherapy dose intensity on cancer patient outcomes. J Natl Compr Canc Netw.

[27] Ramsey SD, McCune JS, Blough DK, McDermott CL, Clarke L, Malin JL, Sullivan SD (2010). Colony-stimulating factor prescribing patterns in patients receiving chemotherapy for cancer. Am J Manag Care.

[28] Sosa R, Li S, Molony JT, Liu J, Stryker S, Collins AJ (2017). Use of prophylactic growth factors and antimicrobials in elderly patients with cancer: a review of the Medicare database. Support Care Cancer.

[29] Gerberich AJ, Attilio MR, Svoboda A (2020). Revisiting same day administration of pegfilgrastim in the age of biosimilars: a review of literature. J Oncol Pharm Pract.

[30] Matera RM, Relias V, Wasif Saif M (2021). Safety and efficacy of same-day administration of pegfilgrastim in patients receiving chemotherapy for gastrointestinal malignancies. Cancer Med J.

[31] Weycker D, Hanau A, Lonshteyn A, Bowers C, Bensink M, Garawin T, Chandler D (2018). Risk of chemotherapy-induced febrile neutropenia with same-day versus next-day pegfilgrastim prophylaxis among patients aged ≥65 years: a retrospective evaluation using Medicare claims. Curr Med Res Opin.

[32] Ma X, Kang J, Li Y, Zhang X (2021). Pegfilgrastim safety and efficacy on the last chemotherapy day versus the next: systematic review and meta-analysis. BMJ Support Palliat Care.

[33] Bartels T, McBride A, AlRawashdh N, Moore L, Persky DO, Abraham I (2021). Same-day pegfilgrastim or pegfilgrastim-cbqv prophylaxis in miniCHOP chemotherapy based regimens for non-Hodgkin lymphoma. J Clin Oncol.

[34] Leiva M, Pennisi A, Harnden KK, Rizzo PC, Mauro LA (2021). The feasibility of same day pegfilgrastim-cbqv administration in breast cancer patients receiving myelosuppressive chemotherapy regimens at a northern Virginia cancer center. J Clin Oncol.

[35] Vraney J, AlRawashdh N, Choi B, Abraham I, McBride A (2021). An institutional evaluation of the safety and efficacy of same-day administration of pegfilgrastim in patients receiving chemotherapy for lung cancer. J Clin Oncol.

[36] Sandschafer D, Choi A, Lewis S, Upchurch T, De Oliveira Brandao C (2022) Assessment of device failures and medication errors with the pegfilgrastim on-body injector in the US and EU. Presented at ISPOR 2022: May 15–18

