# Supplementary material for: A prospective study to evaluate febrile neutropenia incidence in patients receiving pegfilgrastim on-body injector vs other choices
Source: Support Care Cancer. 2022 Jun 22;30(10):7913–22. doi: 10.1007/s00520-022-07226-9 (PMC9216302; doi:10.1007/s00520-022-07226-9)
Supplement: Supplementary file 1 — Supplementary file1 (DOCX 114 KB) [file 520_2022_7226_MOESM1_ESM.docx]

**Supplementary Information**

**Title:** A Prospective Study to Evaluate Febrile Neutropenia Incidence in Patients Receiving Pegfilgrastim On-body Injector vs Other Choices

**Journal name**: *Supportive Care in Cancer*

Robert M Rifkin, Jeffrey Crawford, Reshma L Mahtani, David C Dale, Mohit Narang, William W MacLaughlin, Chanh Huynh, Prasad L Gawade, Sandra Lewis, Lucy DeCosta, Tatiana Lawrence, Rajesh Belani

**Corresponding author:** Robert M Rifkin, MD, FACP, Medical Director Biosimilars, Associate Chair, US Oncology Hematology Research, Rocky Mountain Cancer Centers – Midtown, 1800 Williams Street, Suite 200, Denver, CO 80218. Email: [Robert.Rifkin@usoncology.com](mailto:Robert.Rifkin@usoncology.com); Phone: (303) 388-4876

**Table S1. Comorbidities**

|  | On-body Injector (*n* = 1624) | Other Physician Choice (*n* = 951) |
| --- | --- | --- |
| Comorbidities – *n* (%) | | |
| Hypertension | 802 (49.4) | 462 (48.6) |
| Diabetes | 316 (19.5) | 180 (18.9) |
| Thyroid disorder | 244 (15.0) | 130 (13.7) |
| Osteoarthritis | 232 (14.3) | 125 (13.1) |
| Anemia | 173 (10.7) | 136 (14.3) |
| Coronary artery disease | 138 (8.5) | 88 (9.3) |
| Chronic obstructive pulmonary disease | 128 (7.9) | 98 (10.3) |
| Rheumatoid arthritis | 55 (3.4) | 23 (2.4) |
| Kidney dysfunction | 48 (3.0) | 43 (4.5) |
| Myocardial infarction | 47 (2.9) | 25 (2.6) |
| Emphysema | 33 (2.0) | 26 (2.7) |
| Congestive heart failure | 32 (2.0) | 21 (2.2) |
| Peptic ulcer disease | 32 (2.0) | 15 (1.6) |
| Peripheral vascular disease | 30 (1.8) | 21 (2.2) |
| Hepatic function disorder | 27 (1.7) | 14 (1.5) |
| Autoimmune thyroiditis | 18 (1.1) | 7 (0.7) |
| Multiple sclerosis | 16 (1.0) | 7 (0.7) |
| Systemic lupus erythematosus | 9 (0.6) | 9 (0.9) |
| Dementia | 6 (0.4) | 5 (0.5) |

**Table S2. Patient Disposition**

|  | All Patients (*N* = 2715) *n* (%) |
| --- | --- |
| Enrollment | 2715 (100.0) |
|  |  |
| Study completion accounting | |
| Patients who completed study | 2444 (90.0) |
| Patients who discontinued study | 271 (10.0) |
| Decision by sponsor | 132 (4.9) |
| Withdrawal of consent from study | 57 (2.1) |
| Death | 44 (1.6) |
| Lost to follow-up | 38 (1.4) |

**Table S3. Demographics and Baseline Characteristics in Patients With Curative and Palliative Treatment Intents**

|  | Curative Intent | | Palliative Intent | |
| --- | --- | --- | --- | --- |
|  | On-body  Injector | Other Physician Choice | On-body  Injector | Other Physician Choice |
|  | (*n* = 1424) | (*n* = 725) | (*n* = 200) | (*n* = 226) |
| Sex – *n* (%) |  |  |  |  |
| Male | 150 (10.5) | 106 (14.6) | 121 (60.5) | 145 (64.2) |
| Female | 1274 (89.5) | 619 (85.4) | 79 (39.5) | 81 (35.8) |
| Age (years) |  |  |  |  |
| Median (IQR) | 61 (51–68) | 60 (50–69) | 69 (61–75) | 68 (60–73) |
| Tumor type – *n* (%) |  |  |  |  |
| Breast | 1163 (81.7) | 560 (77.2) | 33 (16.5) | 20 (8.8) |
| Non-Hodgkin lymphoma | 196 (13.8) | 126 (17.4) | 40 (20.0) | 51 (22.6) |
| Lung | 48 (3.4) | 32 (4.4) | 84 (42.0) | 100 (44.2) |
| Prostate | 17 (1.2) | 7 (1.0) | 43 (21.5) | 55 (24.3) |
| ECOG performance status – *n* (%) |  |  |  |  |
| 0 or 1 | 1387 (97.4) | 711 (98.1) | 174 (87.0) | 194 (85.8) |
| ≥2 | 28 (2.0) | 12 (1.7) | 22 (11.0) | 32 (14.2) |
| Missing | 9 (0.6) | 2 (0.3) | 4 (2.0) | 0 (0.0) |
| Number of comorbidities – *n* (%) |  |  |  |  |
| >2 | 254 (17.8) | 124 (17.1) | 71 (35.5) | 71 (31.4) |
| ≤2 | 1170 (82.2) | 601 (82.9) | 129 (64.5) | 155 (68.6) |
| History of any other malignancy^a^ – *n* (%) |  |  |  |  |
| Yes | 67 (4.7) | 53 (7.3) | 30 (15.0) | 42 (18.6) |
| No | 1357 (95.3) | 672 (92.7) | 170 (85.0) | 184 (81.4) |
| Antibiotic use prior to initiation of chemotherapy – *n* (%) |  |  |  |  |
| Yes | 113 (7.9) | 89 (12.3) | 18 (9.0) | 21 (9.3) |
| No | 1311 (92.1) | 636 (87.7) | 182 (91.0) | 205 (90.7) |
| Prior surgery^b^– *n* (%) |  |  |  |  |
| Yes | 1144 (80.3) | 484 (66.8) | 106 (53.0) | 117 (51.8) |
| No | 280 (19.7) | 241 (33.2) | 94 (47.0) | 109 (48.2) |
| Prior chemotherapy^b^ – *n* (%) |  |  |  |  |
| Yes | 4 (0.3) | 3 (0.4) | 3 (1.5) | 5 (2.2) |
| No | 1420 (99.7) | 722 (99.6) | 197 (98.5) | 221 (97.8) |
| Prior radiotherapy^b^ – *n* (%) |  |  |  |  |
| Yes | 26 (1.8) | 9 (1.2) | 9 (4.5) | 15 (6.6) |
| No | 1398 (98.2) | 716 (98.8) | 191 (95.5) | 211 (93.4) |

*ECOG* Eastern Cooperative Oncology Group; *IQR* interquartile range.

^a^Excluding nonmelanoma skin cancer.

^b^Within 6 months prior to study enrollment.

**Table S4. Febrile Neutropenia Risk and Chemotherapy Regimens in Patients With Curative and Palliative Treatment Intents**

|  | Curative Intent | | Palliative Intent | |
| --- | --- | --- | --- | --- |
|  | On-body Injector | Other Physician Choice | On-body Injector | Other Physician Choice |
|  | (*n* = 1424) | (*n* = 725) | (*n* = 200) | (*n* = 226) |
| FN risk of chemotherapy regimen – *n* (%) |  |  |  |  |
| High | 1063 (74.6) | 479 (66.1) | 16 (8.0) | 14 (6.2) |
| Intermediate | 361 (25.4) | 246 (33.9) | 184 (92.0) | 212 (93.8) |
| Chemotherapy regimen – *n* (%) |  |  |  |  |
| High risk for FN (>20%) |  |  |  |  |
| TC | 566 (39.7) | 249 (34.3) | 7 (3.5) | 5 (2.2) |
| TCHP | 384 (27.0) | 176 (24.3) | 8 (4.0) | 6 (2.7) |
| TCH | 81 (5.7) | 32 (4.4) | 1 (0.5) | 0 (0.0) |
| TAC | 23 (1.6) | 8 (1.1) | 0 (0.0) | 1 (0.4) |
| R-da EPOCH | 8 (0.6) | 13 (1.8) | 0 (0.0) | 2 (0.9) |
| Dose-adjusted EPOCH | 1 (< 0.1) | 0 (0.0) | 0 (0.0) | 0 (0.0) |
| R-ICE | 0 (0.0) | 1 (0.1) | 0 (0.0) | 0 (0.0) |
| Intermediate risk for FN (10%–20%) |  |  |  |  |
| R-CHOP | 149 (10.5) | 81 (11.2) | 14 (7.0) | 8 (3.5) |
| AC | 60 (4.2) | 44 (6.1) | 3 (1.5) | 3 (1.3) |
| AC→T | 46 (3.2) | 21 (2.9) | 1 (0.5) | 0 (0.0) |
| Carboplatin and paclitaxel | 32 (2.2) | 19 (2.6) | 27 (13.5) | 36 (15.9) |
| Bendamustine and rituximab | 29 (2.0) | 30 (4.1) | 24 (12.0) | 41 (18.1) |
| Docetaxel | 16 (1.1) | 12 (1.7) | 43 (21.5) | 59 (26.1) |
| Etoposide and carboplatin | 11 (0.8) | 8 (1.1) | 59 (29.5) | 62 (27.4) |
| CHOP | 8 (0.6) | 1 (0.1) | 2 (1.0) | 0 (0.0) |
| Cisplatin and docetaxel | 4 (0.3) | 4 (0.6) | 0 (0.0) | 0 (0.0) |
| CMF classic | 2 (0.1) | 22 (3.0) | 0 (0.0) | 0 (0.0) |
| Cisplatin and etoposide | 2 (0.1) | 3 (0.4) | 0 (0.0) | 0 (0.0) |
| TH | 1 (< 0.1) | 1 (0.1) | 4 (2.0) | 0 (0.0) |
| Paclitaxel | 1 (< 0.1) | 0 (0.0) | 2 (1.0) | 2 (0.9) |
| Cabazitaxel | 0 (0.0) | 0 (0.0) | 4 (2.0) | 1 (0.4) |
| Carboplatin nab-paclitaxel | 0 (0.0) | 0 (0.0) | 1 (0.5) | 0 (0.0) |

*AC* doxorubicin, cyclophosphamide; *AC→T* doxorubicin, cyclophosphamide→docetaxel; *CHOP* cyclophosphamide, doxorubicin, vincristine, prednisone; *CMF* cyclophosphamide, methotrexate, fluorouracil; *EPOCH* etoposide, prednisone, vincristine, cyclophosphamide, doxorubicin; *FN* febrile neutropenia; *ICE* ifosfamide, carboplatin, etoposide; *R* rituximab; *R-da EPOCH* rituximab, dose-adjusted etoposide, prednisone, vincristine, cyclophosphamide, doxorubicin; *TAC* docetaxel, doxorubicin, cyclophosphamide; *TC* docetaxel, cyclophosphamide; *TCH* docetaxel, carboplatin, trastuzumab; *TCHP* docetaxel, carboplatin, trastuzumab, pertuzumab; *TH* docetaxel, trastuzumab.

**Table S5. Demographics, Baseline Characteristics, and Febrile Neutropenia Risk in Patients Who Received Pegfilgrastim OBI and Did Not Receive G-CSF**

|  | On-body  Injector (*n* = 1624) | No G-CSF in All Cycles (*n* = 228) |  |
| --- | --- | --- | --- |
| Sex – *n* (%) | | | |
| Male | 271 (16.7) | 109 (47.8) |  |
| Female | 1353 (83.3) | 119 (52.2) |  |
| Age (years) | | | |
| Median (IQR) | 62.0 (52.0–70.0) | 66.0 (58.0–71.0) |  |
| ECOG performance status – *n* (%) |  |  |  |
| 0 or 1 | 1561 (96.1) | 212 (93.0) |  |
| ≥2 | 50 (3.1) | 15 (6.6) |  |
| Missing | 13 (0.8) | 1 (0.4) |  |
| Number of comorbidities – *n* (%) |  |  |  |
| >2 | 325 (20.0) | 60 (26.3) |  |
| ≤2 | 1299 (80.0) | 168 (73.7) |  |
| History of any other malignancy^a^ – *n* (%) |  |  |  |
| Yes | 97 (6.0) | 25 (11.0) |  |
| No | 1527 (94.0) | 203 (89.0) |  |
| Antibiotic use prior to initiation of chemotherapy – *n* (%) |  |  |  |
| Yes | 61 (3.8) | 6 (2.6) |  |
| No | 1563 (96.2) | 222 (97.4) |  |
| Prior surgery^b^– *n* (%) |  |  |  |
| Yes | 1250 (77.0) | 162 (71.1) |  |
| No | 374 (23.0) | 66 (28.9) |  |
| Prior chemotherapy^b^ – *n* (%) |  |  |  |
| Yes | 7 (0.4) | 3 (1.3) |  |
| No | 1617 (99.6) | 225 (98.7) |  |
| Prior radiotherapy^b^ – *n* (%) |  |  |  |
| Yes | 35 (2.2) | 9 (3.9) |  |
| No | 1589 (97.8) | 219 (96.1) |  |
| Intent of treatment – *n* (%) |  |  |  |
| Curative | 1424 (87.7) | 119 (52.2) |  |
| Palliative | 200 (12.3) | 109 (47.8) |  |
| FN risk of chemotherapy regimen – *n* (%) |  |  |  |
| High | 1079 (66.4) | 32 (14.0) |  |
| Intermediate | 545 (33.6) | 196 (86.0) |  |

*ECOG* Eastern Cooperative Oncology Group; *FN* febrile neutropenia; *G-CSF* granulocyte colony-stimulating factor; *IQR* interquartile range.

^a^Excluding nonmelanoma skin cancer.

^b^Within 6 months prior to study enrollment.

**Table S6. Top 5 Adverse Events in the OBI Group^a^**

| Preferred Term | On-body Injector (*n* = 1624) *n* (%) | Other Physician Choice (*n* = 935) *n* (%) |
| --- | --- | --- |
| Anemia | 332 (20.4) | 142 (15.2) |
| Bone pain | 283 (17.4) | 125 (13.4) |
| Arthralgia | 134 (8.3) | 59 (6.3) |
| Neutropenia | 123 (7.6) | 112 (12.0) |
| Pyrexia | 115 (7.1) | 52 (5.6) |

^a^Adverse events known to occur with chemotherapy were not captured.

**Fig. S1** Study schema. The observation period is from screening/enrollment visit 1/cycle 1 through visit 4 on day 1 of each subsequent chemotherapy cycle. A chemotherapy cycle can range from 3 to 4 weeks in duration.

**
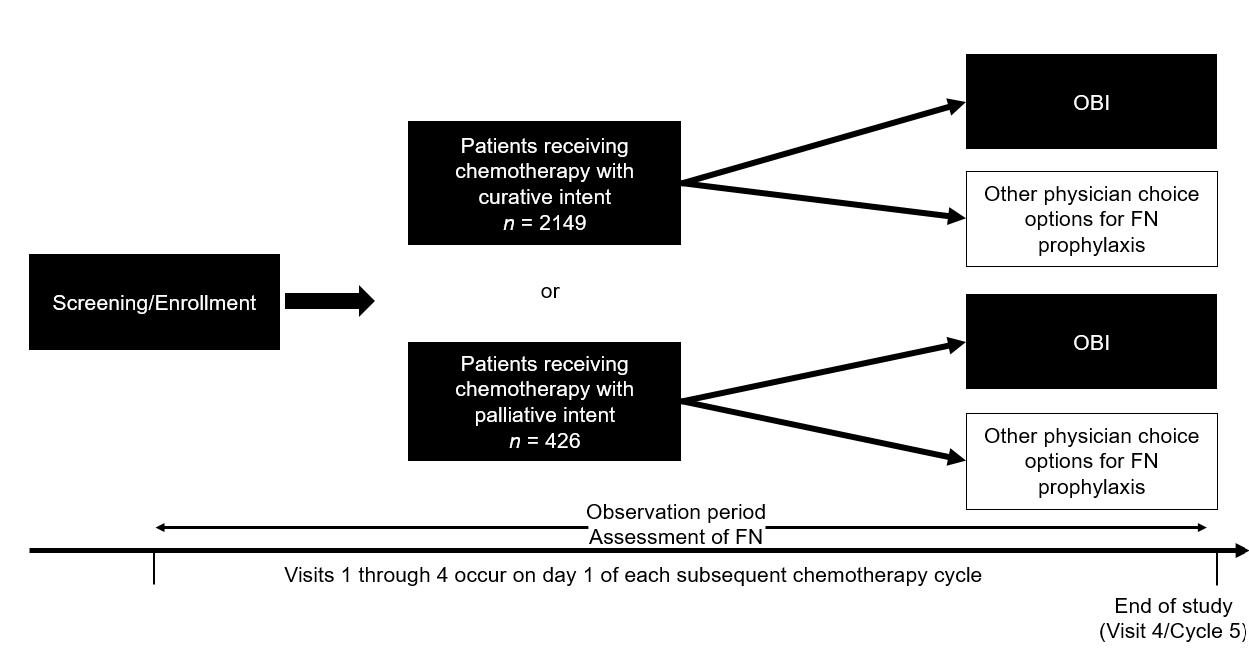
**

*FN* febrile neutropenia; *OBI* on-body injector.

**Fig. S2** Incidence of FN for subgroups. (a) FN incidence for OBI versus Other in patients receiving ≥1 cycle of G-CSF, (b) FN incidence by treatment intent, and (c) FN incidence by tumor type. Error bars denote 95% CIs.

*CI* confidence interval; *FN* febrile neutropenia; *G-CSF* granulocyte colony-stimulating factor; *NHL* non-Hodgkin lymphoma; *OBI* on-body injector; *Other* other physician choice options.
